# Study of Stress Granule Core Protein AtUBP1b Phosphorylation *In Vitro*

**DOI:** 10.3390/plants14203191

**Published:** 2025-10-17

**Authors:** Anna S. Nizkorodova, Valeriy Y. Kislitsin, Andrey V. Zhigailov, Arman T. Kulyyassov, Leila M. Nadirova, Gulshan E. Stanbekova, Bulat K. Iskakov

**Affiliations:** 1 M. Aitkhozhin Institute of Molecular Biology and Biochemistry, Almaty 050012, Kazakhstana.zhigailov@imbb.org.kz (A.V.Z.); leila.nadirova@gmail.com (L.M.N.); gulshanst@yahoo.com (G.E.S.); bulat.iskakov2@gmail.com (B.K.I.); 2 National Center of Biotechnology, Astana 010000, Kazakhstan; akulyyasov@gmail.com

**Keywords:** stress granules, AtUBP1b, phosphorylation, RNA binding protein, gel-shifting

## Abstract

Stress granules (SGs) are dynamic membrane-less structures assembled in response to stress. The formation of stress granules in plants is poorly understood, especially the mechanism of mRNA recruitment. The problem of the specificity of mRNA interaction with stress granule proteins is unexplored. Oligouridylate binding protein 1B (UBP1B) is considered as the core element of plant SGs. In this study, we expressed the AtUBP1b protein from *Arabidopsis thaliana* in *E. coli* cells. Mass spectroscopic analysis showed that the AtUBP1b protein expressed in *E. coli* cells is phosphorylated at serine, threonine, and tyrosine residues. We also performed a *de novo* phosphorylation reaction in wheat germ extracts with the addition of radioactively labeled phosphorus and showed AtUBP1b phosphorylation in plant extracts. We hypothesized that phosphorylation or dephosphorylation of AtUBP1b in plant cells is a signal for protein binding to RNA. The purified protein was tested for its ability to bind to mRNA *in vitro*. In gel-shifting assays we demonstrated that AtUBP1b protein binds specifically to 5′-untranslated regions (5′UTR) of mRNA. When AtUBP1b was added to a cell-free wheat germ translation system, it exerted different effects on protein synthesis. We showed that AtUBP1b had a significant inhibitory effect on the expression of mRNAs containing 5′UTRs that were shown to bind to the protein in the gel-shifting reaction.

## 1. Introduction

Stress granules (SGs) have been studied by the scientific community for decades and much has been learned about their composition and formation mechanism. But the main question—the function and reason for the formation of SGs—remains unanswered.

The function of SGs was considered to be the rapid and specific removal of a large amount of mRNA from biosynthesis processes in the cell, which allows the latter to translate only those proteins that are necessary for survival. At the same time, after the end of stress, the SGs also quickly disband and mRNA and 48S pre-initiation complexes are introduced into the biosynthesis process. However, data from recent studies show that the amount of mRNA removed during stress in the SGs is in principle insignificant—no more than 10% of the total cellular mRNA [1]. It has also been shown that the formation of the SGs does not have a significant effect on the level of global translation in the cell [2,3]. At the same time, a number of studies indicate a direct connection between SG proteins, in particular TIA-1/TIAR, and specific inhibition of translation through the binding of these proteins to the 3′UTR of a number of translation initiation factors [4,5], as well as to the 5′TOP (5′-terminal oligopyrimidine tracts) of the mRNA of a number of biosynthesis factors [6].

It is believed that the process of SG formation is based on the phenomenon of liquid–liquid phase separation, which is similar to coacervate droplet formation [7,8]. Under stress conditions, translation in a eukaryotic cell almost completely stops, which leads to the dissolution of polysomes and the accumulation of both free mRNA and 43S and 48S preinitiation complexes in the cytoplasm, which leads to the initial local concentration of RNA–protein complexes and subsequent phase separation. At some point, the TIA-1/TIAR and G3BP1 proteins (for animal organisms), which are mandatory for all SGs as core proteins, join the process. It has been shown that in the absence of SG core proteins, the formation of the latter is impossible [9], and specific core proteins are required for each type of stress [2]. It has also been shown that the expression level of some SG proteins changes insignificantly during stress [10].

In addition, the extremely short time of SG formation should be taken into account: within 10 min after the onset of stress, stress granules appear in the cytoplasm in *Arabidopsis* [11] and within 10–15 min in animal cells and yeast [12]. Thus, the signal (trigger) that causes RBP proteins to participate in SG formation remains unknown, since outside of stress conditions, these proteins are found in a free form both in the cytoplasm and in the nucleus. It has been suggested [4] that during stress, RBPs can be phosphorylated or modified in some other way, which causes their aggregation and subsequent SG formation. How SGs are subsequently destroyed and preinitiation complexes are released is not exactly known. It is believed that in this process, along with heat shock proteins, RNA helicases and cytoskeletal proteins, such modifications of SG proteins as sumoylation [13] and ubiquitination [14] are also involved. At the same time, for plant organisms, even these minimal data on the processes of the formation and destruction of SGs are absent [7].

Taking into account all of the above, we put forward the following hypothesis for the formation of SGs. We assumed that a certain number of stress granule core proteins, which are in the “non-stress” mode, are constantly present in the cell cytoplasm. In this form, core proteins do not bind mRNA and do not aggregate with each other. When stress conditions arise, core proteins, or some of these proteins, undergo modification, due to which they acquire the ability to specifically bind to definite sequences of mRNA, which is also released from polysomes in large quantities. And in the form of RNA-bound complexes, SG proteins begin to aggregate with each other, which leads to the formation of SGs. This part of the hypothesis is supported by the study by Loughlin et al. [15], which showed that phase separation and fibril formation of the TIA-1 protein is enhanced by binding of the protein to single-stranded RNA or DNA. We considered phosphorylation of the SG core proteins as a possible modification in response to stress.

The proposed hypothesis was investigated on the AtUBP1b (Oligouridylate binding protein) protein, which is one of the marker proteins of stress granules in Arabidopsis. This protein is a plant analogue of the animal protein TIA-1 [16], for which phosphorylation was shown during cell apoptosis [17]. Phosphorylation of TIA1 in cancer cells was also shown at several serine residues, as well as at one threonine and one tyrosine [18].

These proteins (TIA-1 and UBP1) contain three RNA-recognition motifs (RRMs), which provide binding specificity to nucleic acids, as well as one C-terminal glutamine-rich prion-like domain (Q-domain), which promotes protein aggregation [9].

We cloned the *AtUbp1b* cDNA gene into a bacterial expression vector and expressed and purified the AtUBP1b protein in preparative quantities. Phosphorylation of the obtained protein at serine and threonine residues was detected using Western blotting with antibodies against *p*-Ser and *p*-Thr. We further demonstrated the presence of AtUBP1b phosphorylation *de novo* in S23 and S100 extracts from wheat germ upon addition of radioactively labeled ATP. Thus, we demonstrated phosphorylation of the studied protein both in *E. coli* cells and in plant extracts. Mass spectrometry of the AtUBP1b protein obtained from bacterial cells gave coverage of 74% and confirmed the presence of phosphorylation at serine, threonine, and tyrosine residues. Next, in a cell-free translation system from wheat germ, we showed an inhibiting effect on the translation of AtUBP1b added to the system in the proportion 5:1 to mRNA. We also demonstrated the specificity of AtUBP1b interaction with mRNA depending on its 5′-untranslated region (UTR) sequence. We showed direct interaction of the partially phosphorylated form of the AtUBP1b protein with several types of 5′UTR using EMSA (electrophoretic mobility shift assay).

## 2. Results

### 2.1. Expression and Purification of AtUBP1b from E. coli

**Figure 1:**
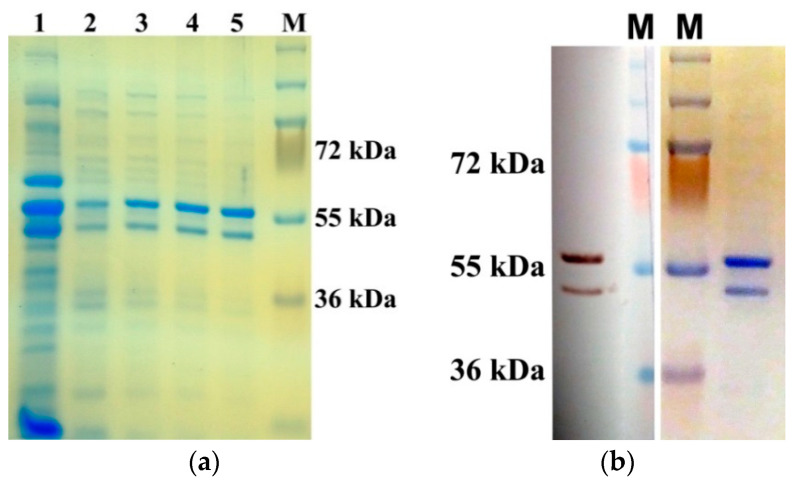
Results of AtUBP1b protein purification from *E. coli* cells. (**a**) PAGE with fractions of protein obtained under native conditions. Lanes 2–5 show protein samples eluted with increasing concentrations of imidazole; lane 1 shows crude lysate of total cellular protein. (**b**) Final protein sample of AtUBP1b, dialyzed twice against TBS after purification: Western blot with anti-His-tag antibodies (on the left), PAGE stained with G-250 (on the right). M—page ruler plus prestained protein ladder (Thermo Fisher Scientific, Waltham, MA, USA).

The AtUBP1b protein has 419 amino acid residues [19], and its calculated molecular mass is 47 kDa. The protein was expressed in *E. coli* cells and purified by affinity chromatography on Ni-NTA agarose under native conditions (Figure 1a).

It should be noted that the AtUBP1b protein was detected on the gel by two bands with a difference in electrophoretic mobility of ~10 kDa. Figure 1b shows the final sample of the AtUBP1b protein (the purity of the preparation was more than 95% according to densitometry), where it is evident that both protein bands are recognized by specific anti-His-tag antibodies. The upper band, which is heavier in electrophoretic mobility, is more distinct. Since the protein was isolated with the addition of phosphatase inhibitors, we assumed that the upper band corresponds to the protein in the phosphorylated form, and the lower one in the dephosphorylated form. To test this assumption, we isolated a mini-preparation of the AtUBP1b protein using the same scheme as the previous preparation, except for the addition of phosphatase inhibitors. The electrophoretic analysis and Western blot (Figure S1 Supplementary) showed that a similar protein preparation is characterized by a multiple increase in the amount of protein of the lower (conditionally dephosphorylated) band.

### 2.2. Determination of AtUBP1b Phosphorylation

**Figure 2:**
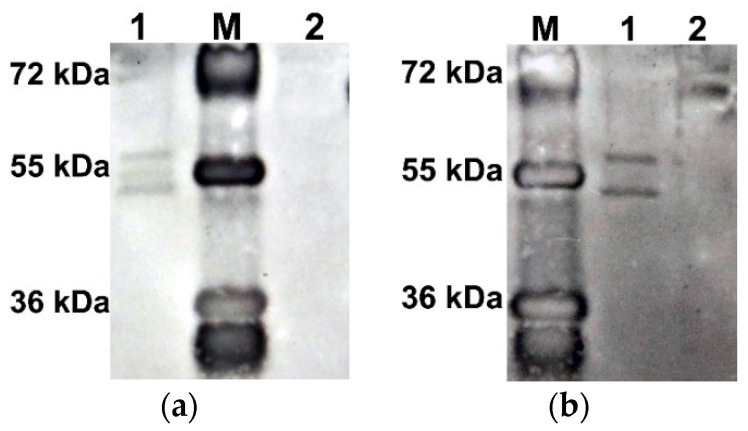
Western blot of AtUBP1b with anti-*p*-Ser antibodies (**a**) and with anti-*p*-Thr antibodies (**b**). Lane 1—AtUBP1b, lane 2—BSA, M—page ruler plus prestained protein ladder (Thermo Scientific). Detection was performed with “Chemiluminescent Peroxidase Substrate-3” (Sigma-Aldrich, St. Louis, MO, USA).

To test the hypothesis that the difference in electrophoretic mobility of the two AtUBP1b protein bands is due to their phosphorylation, Western blot analysis was performed with antibodies to phosphorylated forms of serine (*p*-Ser) and threonine (*p*-Thr). The results (Figure 2) showed that both bands are phosphorylated, both at serine and threonine residues.

**Figure 3:**
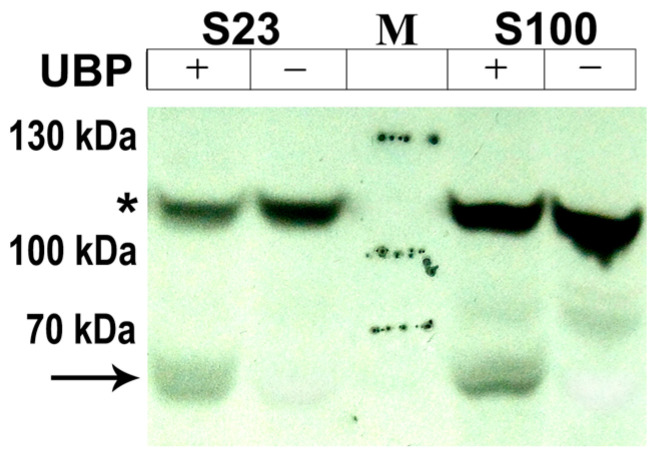
*De novo* phosphorylation of AtUBP1b in S23 (suitable for *in vitro* translation) and S100 extracts (without ribosomes) from wheat germ on X-ray film. “UBP+” lanes indicate wheat germ extracts to which AtUBP1b was added; “UBP−” lanes show wheat germ extracts with no addition of protein; arrow indicates phosphorylated AtUBP1b; asterisk—one of the common phosphorylated proteins from the wheat germ extracts; M—page ruler plus prestained protein ladder (Thermo Fisher Scientific, Waltham, MA, USA).

Since the AtUBP1b protein was expressed in *E. coli* cells, there was a possibility that the phosphorylation we observed was an artifact and was characteristic only of bacterial cells. In order to test the fundamental possibility of AtUBP1b protein phosphorylation in plant organisms, we performed a *de novo* phosphorylation reaction in wheat germ extracts with the addition of radioactively labeled phosphorus ([γ-^33^P]ATP). We used both the S23 extract, which is used for *in vitro* protein biosynthesis [20], and the S100 extract, which is a ribosome-less version of S23. Our results (Figure 3) showed the presence of AtUBP1b protein phosphorylation in plant extracts.

The presence of an additional band with the expected electrophoretic mobility in samples with the addition of AtUBP1b indicated phosphorylation of the protein in the plant extract. But this fact did not clarify the question of which amino acid residues are phosphorylated. Based on the fact that a certain group of kinases in prokaryotic cells, in particular *E. coli*, exhibits an activity similar to that of eukaryotic kinases [21], we decided that in plant cells, protein phosphorylation can occur at the same phosphorylation sites as in *E. coli* cells. Thus, it is known that *E. coli* cells contain a kinase that exhibits the activity of protein kinase C of eukaryotic organisms [22,23]. This gave us reason to assume that the phosphorylation sites for serine and threonine residues may be identical for *E. coli* and eukaryotic organisms.

**Figure 4:**
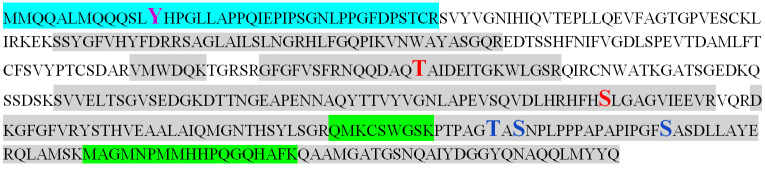
Mass spectrometry analysis of AtUBP1b protein. The combined results for both AtUBP1b bands, which differ in electrophoretic mobility, are shown: the sequence read for both bands is shown in gray, for the lower band only in blue, and for the upper band only in green. Phosphorylated amino acid residues are shown in bold, enlarged font: red—phosphorylation was shown for both bands, purple—for the lower band only, and blue—for the upper band only.

For this reason, we performed mass spectrometry of the AtUBP1b protein obtained as a result of bacterial expression. AtUBP1b bands with different electrophoretic mobility (upper and lower) were analyzed separately. The summarized results of the mass spectroscopic analysis are shown in Figure 4: three phosphorylated serine residues (positions 269, 330, 345), two threonine (182, 328), and one tyrosine (13) were detected. The readout rate was 62% for the upper band of the AtUBP1b protein, 67% for the lower band, and 74% for the combined readout of both bands.

### 2.3. AtUBP1b Induced Sequence-Specific Gel Shifting of RNA

We decided to test our initial assumption that AtUBP1b protein binds to mRNA specifically using a gel-shifting reaction. Binding of UBP1 family proteins to U-rich sequences was initially demonstrated *in vitro* in nuclear extracts of *N. plumbaginifolia* by UV cross-linking with synthetic introns [24]. The following studies of the possibility of plant UBP1 binding to RNA also concerned introns: Lambermon et al., using UV cross-linking, demonstrated the preferential binding of NpUBP1 to U-rich synthetic introns *in vitro* [16]. However, the specificity of the binding of these proteins to RNA as core proteins of stress granules has not been studied yet.

It is known that proteins of the UBP1 family have been isolated from plants mainly in association with introns, 5′UTR or 3′UTR [16]. Since we planned *in vitro* translation for further study of RNAs that would bind to AtUBP1b, the intron sequences were not suitable for us. As for the 3′UTR, it was shown [4] that plant proteins of the UBP1 family preferentially associate with RNAs with U-rich 3′-untranslated regions (3′UTRs) when immunoprecipitated from plants grown under normal conditions. But under stress conditions, proteins of the UBP1 family are isolated together with nuclear poly (A)^+^ RNA but no specificity of RNA-binding was established [4]. Since we are investigating AtUBP1b protein binding to RNA in response to stress, the logical and only choice was 5′UTR sequences.

As RNA matrices for binding reaction, we selected a number of 5′UTRs of predominantly plant viruses containing different amounts of uridine. Table 1 presents the sequences and the corresponding amounts of uridine. We selected the plant virus sequences in order to subsequently use them as part of mRNA in the *in vitro* translation system. Since almost all of the selected sequences are translation enhancers, the effect of AtUBP1b on the translation level will be more accurate on such mRNAs. The 5′UTR size varied from 182 nt (potato virus Y) to 39 nt (εII [25]).

We studied the binding of AtUBP1b to the 5′UTR of such viruses as potato virus Y (PVY), potato virus M (PVM), tobacco mosaic virus (TMV, its 5′UTR is designated as Ω-element), and tobacco etch virus (TEV). We also used two prokaryotic translation enhancers—ipsilon-element (εII) and 5′UTR of cold-shock protein A of *E. coli* (5′CspA). In addition, PVY 5′UTR was represented by a number of incomplete sequences—5′I (PVY_1–132_), 5′F (PVY_1–65_), 5′C (PVY_66–132_), and 5′720 (PVY_133–182_)—which differed both in uridine content and in the presence of secondary structures in RNA (the location of these partial sequences in the 5′UTR of PVY is shown in Table 1).

**Figure 5:**
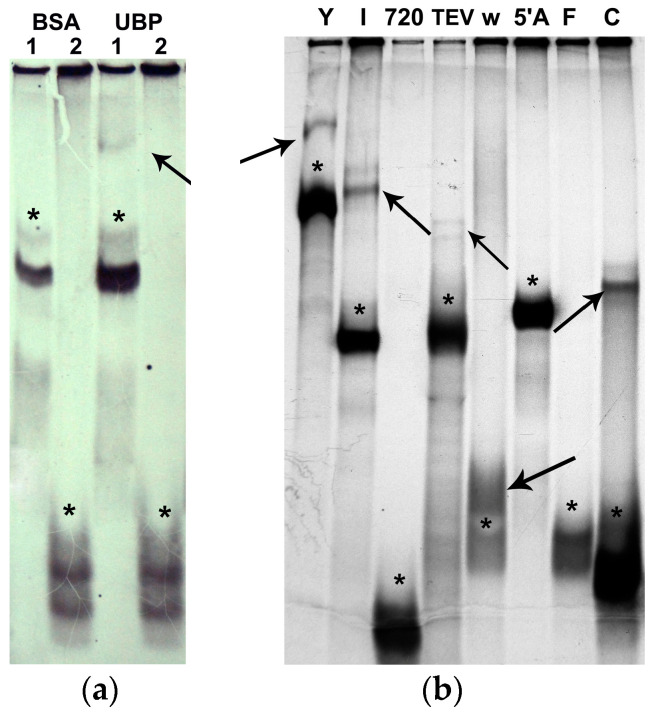
Results of gel-shifting assay in 10% PAGE on X-ray film. (**a**) Gel shifting of 5′PVM (lane 1) and 5′εII (lane 2) with addition of BSA or AtUBP1b proteins; (**b**) gel shifting of Y (5′PVY), I (PVY1-132), F (PVY1-65), C (PVY66-132), 720 (PVY133-182), TEV (5′TEV), w (Ω-element), 5′A (5′CspA) with addition of AtUBP1b. Arrows show RNA–protein complexes; asterisk—RNA that was not bound.

Initial experiments were aimed at establishing the protein to RNA ratio at which binding and detectable gel shifting could occur. Figure 5a shows the results obtained with an RNA to protein ratio of 1:5 (by molarity). Further experiments were performed at this RNA to protein ratio. All binding reactions were repeated at least twice. The final results of the gel-shifting study are shown in Figure 5b.

The obtained results on AtUBP1b binding to RNA led to a number of interesting observations. We concluded that the amount of uridine does not matter when AtUBP1b binds to 5′UTR. 5′CspA did not bind to the protein, while 5′Ω did, although the uridine content in the sequences is the same—26%, as well as for the 5′720 and 5′C sequences, which have 32% and 34%, respectively. The length of the sequence also cannot be decisive (possibly with the exception of the shortest 5′εII)—the sequences 5′F and 5′C are of the same length (5′F does not bind), and 5′CspA and 5′TEV are almost the same length (5′CspA does not bind).

We analyzed all the studied RNAs in the RNAstructure ver.5.4 program (Mathews Lab, Rochester, NY, USA) and came to the conclusion that, perhaps, the main condition for binding of AtUBP1b protein to the 5′UTR of RNA is the presence of secondary structures in the form of sufficiently extended double-stranded regions. In Figure S2 (Supplementary), we present the secondary structures obtained in the program. It should be noted that the sequences 5′720 and 5′F, which did not bind to AtUBP1b, did not form secondary structures at all. The 5′εII sequence, which also did not bind to the AtUBP1b protein, is likely too short to form a bond, since it formed a short double-stranded hairpin, but with a lack of free energy (−0.4 kcal/mol), i.e., it was too unstable. The opposite was observed for the 5′CspA sequence, which also did not bind to the protein. In this case, virtually the entire sequence formed one extended hairpin with a very low free energy (−27.9 kcal/mol), i.e., the structure was so stable that it could not interact with the protein. All other RNAs had much higher free energy; even the secondary structure of the longest 5′PVY was of −11 kcal/mol.

### 2.4. AtUBP1b Influence on In Vitro Translation

**Figure 6:**
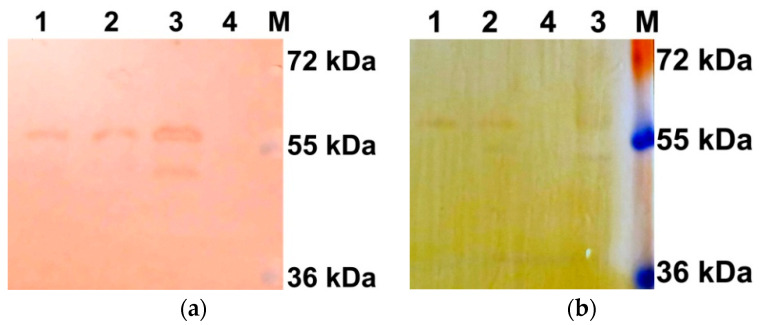
Western blot of AtUBP1b after dephosphorylation with lambda-phosphatase: (**a**) *p*-Ser antibodies and (**b**) *p*-Thr antibodies. Lane 1—AtUBP1b dephosphorylated with double amount of λ-phosphatase; 2—AtUBP1b dephosphorylated with standard amount of λ-phosphatase; 3—original non-dephosphorylated AtUBP1b; lane 4—BSA treated with standard amount of λ-phosphatase; M—page ruler plus prestained protein ladder (Thermo Fisher Scientific, Waltham, MA, USA). Detection was performed with metal-enhanced DAB substrate kit (Thermo Fisher Scientific, Waltham, MA, USA).

To determine the effect of the phosphorylated and dephosphorylated forms of AtUBP1b on translation *in vitro*, we performed a dephosphorylation reaction of the protein with λ-phosphatase. The protein obtained after this was analyzed by Western blot using antibodies to phosphorylated forms of serine (*p*-Ser antibodies) and threonine (*p*-Thr antibodies). According to the results obtained (Figure 6), dephosphorylation of the protein was incomplete, even with a twofold increase in the amount of enzyme—the upper, less electrophoretically mobile band remained phosphorylated at both the serine and threonine residues.

Nevertheless, we decided to use the obtained partially dephosphorylated protein for the *in vitro* translation reaction. In order to take into account the effect on translation of λ-phosphatase itself, the control protein BSA was also treated with this enzyme. Thus, we used two pairs of protein preparations: AtUBP1b and BSA without the addition of λ-phosphatase and the same proteins treated with λ-phosphatase. The *in vitro* obtained mRNAs encoding the GUS protein and differing in their 5′ and 3′UTRs were used as matrices. We selected constructs with 5′UTR, for which binding to AtUBP1b in the gel-shifting reaction has been previously shown—Ω (5′UTR of tobacco mosaic virus), 5′PVY (5′UTR of potato virus Y), and 5′TEV (5′UTR of tobacco etch virus)—as well as a construct containing ‘element 720’ (last 50 *n* of 5′PVY), which did not show binding to AtUBP1b. We used tobacco mosaic virus (3′TMV) and potato virus Y (3′PVY) sequences as 3′UTR.

**Figure 7:**
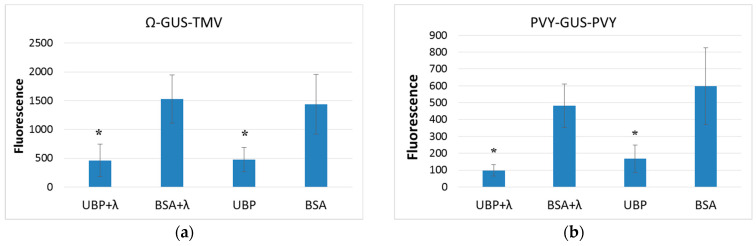

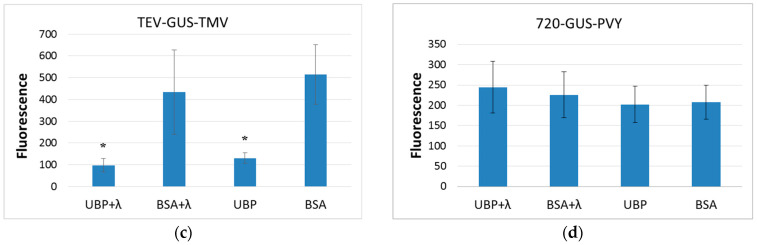
Influence of AtUBP1b on different mRNA translation in the wheat germ extract. The protein expression level was assessed by the activity of GUS enzyme included in the constructs: (**a**) containing 5′- and 3′UTR of tobacco mosaic virus; (**b**) 5′- and 3′UTR of potato virus Y; (**c**) 5′UTR of tobacco etch virus and 3′UTR of tobacco mosaic virus; (**d**) 5′-‘element 720’ and 3′UTR of potato virus Y. Protein AtUBP1b or BSA (as control) was added at a ratio of 5:1 to mRNA. Proteins dephosphorylated by lambda-phosphatase are marked as ‘UBP+λ’ and ‘BSA+λ’. Translation level values significantly different from the control (BSA) are marked with an asterisk; the number of repeats is 4–5 (n).

We assessed the level of protein biosynthesis in a cell-free system from wheat germ by the activity of the resulting GUS protein. We used fluorescent MUG (4-Methylumbelliferyl-ß-D-glucuronide) as a substrate [26]. The levels of GUS expression are shown in Figure 7.

We performed statistical analysis on the obtained data; the results are presented in Table S1 (Supplementary). For all types of mRNA, no significant difference in translation level between control samples ‘BSA+λ’ and ‘BSA’ was observed. Thus, we concluded that the presence of lambda-phosphatase in the samples does not have a significant effect on the functioning of the translation apparatus.

Based on the obtained data, no effect of AtUBP1b protein on ‘720-GUS-PVY’ mRNA during translation was observed (Figure 7d), in either the partially dephosphorylated form (‘UBP+λ’) or the phosphorylated form (‘UBP’), which was in line with our expectations, since no gel-shifting effect with 5′UTR, represented by the ‘720’ sequence, was observed. Also no effect of the presence of the PVY 3′UTR was observed, despite the fact that this element is a U-rich sequence with a uridine content of 37.8% (Table S1, Supplementary). Based on previous studies [4], we expected at least a moderate but statistically significant effect of AtUBP1b protein on the translation of mRNA containing the U-rich 3′UTR.

The effect of AtUBP1b protein on ‘Ω-GUS-TMV’ mRNA during translation was significant both for dephosphorylated (+λ) and phosphorylated proteins (Figure 7a). The translation level for ‘UBP+λ’ was 30% of the translation level for ‘BSA+λ’ (*d* = 2.98), and for ‘UBP’ to ‘BSA’, it was 33% (*d* = 2.42). The degree of AtUBP1b phosphorylation did not affect the translation level, as no statistically significant difference was observed for ‘UBP+λ’ and ‘UBP’. It should be noted that the AtUBP1b inhibitory effect on translation took place despite the fact that the uridine content in both the 5′UTR (Ω) and 3′UTR (TMV) was standard—25.51% and 27.64%, respectively, which leads us to the conclusion that AtUBP1b binding to RNA depends not so much on the amount of uridine, but on the RNA sequence itself.

The ‘UBP+λ’ sample had the greatest effect on the translation of ‘PVY-GUS-PVY’ mRNA (Figure 7b), where the comparative expression level was 20% of ‘BSA+λ’ (*d* = 4.097). For proteins without added lambda-phosphatase, the ‘UBP’ expression level was 28% of ‘BSA’ (*d* = 2.525). It should also be noted that for this mRNA, there was a difference in the effect on the translation of phosphorylated (‘UBP’) and partially dephosphorylated (‘UBP+λ’) forms of the protein, but the statistical significance of these data was incomplete. Thus, the significance level (α) was within acceptable limits, but the power of the test (1-β) was insufficient—22%. However, a preliminary conclusion can be made based on these data: the partially dephosphorylated form (‘UBP+λ’) inhibits translation more strongly than the phosphorylated form (‘UBP’)—the comparative expression level for ‘UBP+λ’ was 58% of that for ‘UBP’ (*d* = 1.085).

A similar pattern of effects on translation was observed for ‘TEV-GUS-TMV’ mRNA (Figure 7c). Dephosphorylated ‘UBP+λ’ inhibited translation by up to 22% (*d* = 2.418) compared to the ‘BSA+λ’ control, while phosphorylated ‘UBP’ inhibited translation by up to 25% (*d* = 3.896). The difference in the effects of dephosphorylated ‘UBP+λ’ and phosphorylated ‘UBP’ forms of the protein on translation was also statistically significant (α), but the test power was insufficient (1-β), which amounted to 6%. And, again, ‘UBP+λ’ inhibited translation more strongly than ‘UBP’ (75% of the translation level of the latter).

## 3. Discussion

The most common post-translational modification of RNA-binding proteins is phosphorylation [18]. There are virtually no data on the phosphorylation of plant stress granule proteins; on the other hand, a wealth of data has been collected on animal analogues of UBP1 family proteins that may well be applicable for AtUBP1b. Phosphorylation of TIA-1, an animal analogue of plant UBP1 family proteins, is most often mentioned in connection with alternative splicing of the Fas receptor [27,28]. It has been shown [27] that the phosphorylation by the Fas-activated Serine/Threonine Kinase (FAST K) form of TIA-1 enhances the interaction of U1 snRNP with suboptimal mRNA splicing sites. However, no change in the degree of binding to mRNA of phosphorylated TIA-1 compared to non-phosphorylated TIA-1 was observed.

Our analysis of previous studies on the AtUBP1b plant analogues showed that when studying the properties of the NpUBP1 protein from *Nicotiana plumbaginifolia* [16], this protein demonstrated electrophoretic mobility characteristic of a protein with a larger mass than the calculated one. The calculated mass of the NpUBP1 protein is 44 kDa, while the mass of this protein in nuclear and cytoplasmic extracts was 50–55 kDa. It should be noted that the NpUBP1 protein sequence shows 75% identity with AtUBP1b. In a study of the UBP1c protein from *Arabidopsis thaliana* [4], an increased molecular mass (by electrophoretic mobility) of ~52 kDa was also recorded instead of the calculated 47 kDa. The authors of these studies did not find an explanation for this effect, and in light of the data obtained in our work, it can be assumed that the ability to phosphorylate may be characteristic of all proteins of the UBP1 family. We demonstrated phosphorylation of the AtUBP1b protein directly in plant extracts from wheat germ by incorporation of radioactively labeled phosphorus into the protein. Considering that the protein was also partially phosphorylated in *E. coli* cells, forming two stable modifications, we came to the conclusion that the kinase (or kinases) performing phosphorylation should be similar in Arabidopsis and *E. coli*.

**Figure 8:**
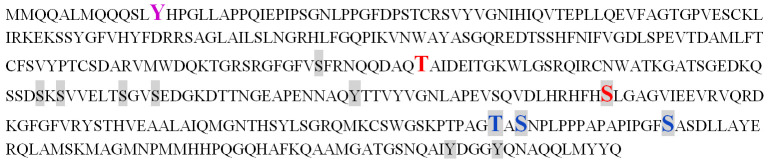
Results of the AtUBP1b protein analysis for the presence of phosphorylation obtained from the MusiteDeep Internet resource. The gray marker indicates the amino acid residues for which the possibility of phosphorylation is predicted on MusiteDeep Internet server. The results of the mass spectrometry for the presence of phosphorylation are shown in bold enlarged font: red—phosphorylation was shown for both bands of the AtUBP1b protein, purple—only for the lower band, blue—only for the upper band.

In addition to the kinases characteristic only for bacteria, kinase activity on serine and threonine residues in *E. coli* cells is also carried out by the Ca^2+^-dependent PKC-like kinase [22,29]. To date, phosphorylation on tyrosine residues in *E. coli* cells has been shown only for the specific bacterial kinase Wzc [30]. Therefore, we assumed that phosphorylation on a tyrosine residue, which was revealed by mass spectrometry, is most likely a bacterial artifact. We analyzed the AtUBP1b protein sequence for possible phosphorylation sites on the MusiteDeep Internet server [31,32]. Figure 8 shows the predicted possible phosphorylation sites for green plant kinases by this resource—four of them match the results of our mass spectrometry. Thus, we can say with a certain degree of confidence that the AtUBP1b protein in the plant organism is phosphorylated at one (or all) of the three matched serine sites and/or one threonine. It is also worth noting that one of the predicted serine phosphorylation sites (217) turned out to be outside the read sequence.

The initial hypothesis of this work, in addition to the phosphorylation of the AtUBP1b protein, was the specificity of its binding (as well as its analogues) to certain elements of mRNA. The results of gel shifting showed that the AtUBP1b protein does indeed have binding specificity towards the 5′UTR of mRNA. Moreover, the binding does not depend on the length of the mRNA sequence, but on its structure, as can be seen in the example of the ‘C’ and ‘F’ sequences (Figure 5), which have the same length but show opposite results in binding to the protein.

For the TIA-1 protein, it was shown [33] that its three RNA recognition motifs (RRMs) have different affinities for RNA sequences. Thus, RRM1 is very weakly involved in binding to RNA, RRM2 is able to interact with both U-rich and UC-rich sequences, and RRM3 promotes protein binding only to UC-rich sequences [34]. And in the study of Cruz-Gallardo et al. [35], it is suggested that due to RRM3, which provides a significant increase in TIA-1 binding to C-rich sequences, it is quite possible for this protein to interact with TOPs (5′ terminal oligopyrimidine tracts) of mRNAs whose translation is repressed under stress. Thus, the possibility of binding of TIA-1 family proteins (and their plant analogs) to the 5′UTR of mRNA has already been discussed.

We hypothesized that the determining factor for AtUBP1b binding to the 5′UTR of mRNA is the presence of a secondary structure in the RNA represented by a relatively stable double-stranded hairpin. The analysis of predicted interactions within the RNA (Figure S2, Supplementary) is consistent with the data obtained in the gel-shifting reaction, with the exception of the 5′CspA sequence, which forms an extended double-stranded hairpin but does not bind to the protein. It is possible that the excessive stability of this structure actually hinders interaction with the protein, as was shown for some isoforms of *SERPINA1* RNAs [36,37].

It is also necessary to take into account the fact that all experiments on binding of 5′UTR to the AtUBP1b protein were carried out with protein, part of which was phosphorylated, part dephosphorylated, and part partially phosphorylated. Therefore, we were unable to establish which particular group of protein molecules participated in the binding reactions, as well as the possibility that the phosphorylation status did not affect the binding of the protein to RNA. In order to determine what effect the phosphorylated protein has in contrast to the dephosphorylated one, we dephosphorylated AtUBP1b using λ-phosphatase. The reaction was partially successful, since partially phosphorylated AtUBP1b (protein with higher electrophoretic mobility—lower band) was completely dephosphorylated, but phosphorylation of the protein with lower electrophoretic mobility (upper band) was preserved, although to a lesser extent. Since the degree of phosphorylation could be assessed only using antibodies to *p*-Ser and *p*-Thr, we were unable to assess tyrosine phosphorylation. The obtained protein sample, in which phosphorylation of protein with higher electrophoretic mobility was absent, was used in *in vitro* translation reactions. It should also be mentioned that in addition to λ-phosphatase, we tried to dephosphorylate AtUBP1b using CIAP and shrimp alkaline phosphatase, but did not obtain any results, since, judging by the Western blot, there was no noticeable difference in protein phosphorylation.

In order to test the effect of AtUBP1b protein binding to mRNA, we conducted experiments on protein translation in a cell-free system from wheat germ. Since protein expression was performed *in vitro*, the initial conditions corresponding to the stress state were obtained in a natural way—all mRNAs at the beginning of the reaction were outside the complexes with ribosomes or translation initiation factors. Against this background, the AtUBP1b protein introduced into the system was able to directly interact with mRNA. Our data showed that AtUBP1b affects protein translation through interaction with the 5′UTR of mRNA. Since we assessed the translation level in comparison with the control reaction, to which BSA was added, the observed effect cannot be explained only by a quantitative increase in the protein level in the cell-free system.

The effect of AtUBP1b on translation was inhibitory, but only for those mRNAs that contained 5′UTRs that showed binding to the protein in gel-shifting reactions—5′PVY, 5′TEV, and 5′Ω. All three 5′UTRs also formed secondary structures with free energies of −11 kcal/mol, −7.9 kcal/mol, and −1.6 kcal/mol, respectively (Figure S2 Supplement). At the same time, 5′-‘element 720’ did not show binding to AtUBP1b or the ability to form a secondary structure. In accordance with our hypothesis, the addition of AtUBP1b to the cell-free translation system did not have any significant effect on the translation level of mRNA containing this 5′UTR.

For all three mRNAs containing 5′UTR binding to AtUBP1b, a significant decrease in the level of protein biosynthesis was shown—up to 20–33% of that of the control. We assume that this is due to the inhibition of translation at the initiation level, since the protein bound to the 5′UTR. The fact that translation is inhibited by AtUBP1b binding to 5′UTR also supports our hypothesis on the mechanism of SG formation.

We propose that protein binds to double-stranded regions of the 5′UTR; thereafter the Q-rich domain (prion-related domain) of protein becomes capable of aggregation with the Q-rich domains of other AtUBP1b proteins also bound to the 5′UTR. The resulting aggregates, containing AtUBP1b and mRNA, are removed from biosynthesis as filaments. Thus, we believe that translation inhibition occurs because such aggregates interfere with ribosome assembly.

To determine the functional impact of AtUBP1b phosphorylation on translation *in vitro*, we generated AtUBP1b protein with a lower degree of phosphorylation due to the action of λ-phosphatase. Promising data were obtained when comparing the effect of AtUBP1b and ‘AtUBP1b+λ’ (protein treated with λ-phosphatase) on translation. Although the level of statistical significance was insufficient due to the low power of the test (1-β), nevertheless, for mRNAs containing 5′PVY and 5′TEV, a greater inhibition of translation was shown with the addition of ‘AtUBP1b+λ’ than with the addition of AtUBP1b. The protein in the ‘AtUBP1b+λ’ sample differed from AtUBP1b in the absence of the partially phosphorylated form (lower band on the gel) and a lower quantity of the phosphorylated form (upper band).

For 5′PVY, the translation level of ‘AtUBP1b+λ’ was 59% of AtUBP1b, while for 5′TEV it was 75%. It should be noted that the significance level (α) was above 95% in both cases (Table S1). These data allow us to draw the preliminary conclusion that the degree of phosphorylation of AtUBP1b affects its ability to inhibit translation. Since inhibition occurs due to AtUBP1b binding to the 5′UTR, we can also assume that the phosphorylation state influences AtUBP1b’s ability to bind to mRNA. This may indicate that AtUBP1b protein binding to mRNA is more likely to occur when it is in a dephosphorylated state.

## 4. Materials and Methods

### 4.1. Cloning of the AtUbp1b cDNA Gene, Expression, and Protein Purification from E. coli

*A. thaliana* total RNA was isolated from 6-week seedlings grown *in vitro*; isolation was performed with TRIzol reagent (Sigma-Aldrich, St. Louis, MO, USA). cDNA was synthesized with M-MLV reverse transcriptase (Thermo Fisher Scientific, Waltham, MA, USA) from oligo (d)T. PCR was carried out with Pfu-polymerase (Promega Corporation, Madison, WI, USA) and specific primers (Fw_Ubp 5′-acggatccatgcagaggttgaagcagcagcag-3′; Rv_Ubp 5′-gcgtcgacaagatcttactggtagtacatgag-3′). PCR product was cloned into pQE-30 (Qiagen, Venlo, The Netherlands) by restriction sites *Bam*HI and *Sal*I under the control of T5-promoter and N-flanking hexa-His-tag.

Protein expression was performed in *E. coli* M15 strain with the addition of 1 mM IPTG for 16 h at RT. Protein purification was performed on Ni-NTA-agarose (Qiagen, Venlo, The Netherlands) under native conditions with increasing gradient of imidazole (up to 300 mM) and the addition of phosphatase inhibitor Coctail C (Santa Cruz Biotechnology, Dallas, TX, USA). The entire extraction procedure was carried out according to the manufacturer’s recommendations for Ni-NTA-agarose (Qiagen, Venlo, The Netherlands). The purity of the obtained fractions was assessed by Western blotting with mouse anti-His-tag antibodies (Santa Cruz Biotechnologies, Dallas, TX, USA) and by staining the gels with colloidal Coomassie. The purest fractions were pooled and dialyzed against TBS at 4 °C for 16 h. The final protein concentration after isolation was 35 mg/mL; protein yield from 100 mL of *E. coli* cell culture was 83 mg. The purified protein was stored at −80 °C in TBS. Protein stability was not studied separately; however, no protein degradation was observed on electrophoresis after repeated freeze–thaw cycles.

### 4.2. Cell-Free In Vitro Translation

S23 extract from wheat germ was prepared according to [20] from wheat Kazakhstanskaya-10. mRNAs were produced by T7-RNA-polymerase (Thermo Fisher Scientific, Waltham, MA, USA) from pBluescriptII matrices carrying gen *uidA* (GUS) and linearized with *Eco*RI. For one reaction of 20 μL, we used 5 μL of S23 extract and 0.5 μg of RNA. Proteins—AtUBP1b and BSA (bovine serum albumin)—were added to the translation system in a 5:1 concentration to mRNA. BSA was added as a control protein. Translation was held for 1 h at 26 °C; to stop the reaction, tubes were put into the ice. The translation level was evaluated by the GUS activity on a fluorescent MUG substrate according to [26] on a TKO-100 fluorometer (Hoefer Scientific Instruments, San Francisco, CA, USA) with absorption at a wavelength of 365 nm and emission at a wavelength of 460 nm.

### 4.3. Protein Phosphorylation In Vitro

For protein phosphorylation we used S23 and S100 extracts from wheat germ. S23 was prepared as mentioned above [20]. The S100 extract was prepared accordingly, with the difference that after the second centrifugation at 23,000× *g*, the supernatant fraction was centrifuged for 2 h at 100,000× *g*. The reaction of 22 μL contained 20 mM tris acetate (pH = 7.5), 2.5 mM magnesium acetate, 90 mM potassium acetate, 1.6 mM DTT, 9 μM ATP, radiolabeled [γ-^33^P]ATP 1.2 μCi, 100 μM of desired protein, and 5 μL wheat germ extract. The reaction lasted for 1 h at 26 °C. The level of *de novo* protein phosphorylation was evaluated by ^33^P inclusion into protein on SDS electrophoresis and subsequent roentgenogram on X-film CP-BU-M (Agfa, Mortsel, Belgium).

### 4.4. Radio-Labeled mRNA Synthesis

Radio-labeled mRNAs were synthesized *in vitro* from the constructions based on plasmid pBluescriptIIKS+ that carry different types of 5′UTR. Plasmid DNAs were restricted by *Nco*I at the AUG site, precipitated and washed in ethanol, and then used in a transcription *in vitro* reaction. *In vitro* transcription was performed using the Thermo Scientific Transcript Aid T7 High Yield Transcription Kit (Thermo Fisher Scientific, Waltham, MA, USA). Each reaction besides the buffer, T7-RNA-polymerase, and NTPs contained 2 μg of linearized DNA template and 1 MBq of [α-^32^P]UTP. The transcription reaction lasted for 2 h at 37 °C with subsequent ethanol precipitation of RNA products. Quantitation of RNA yields was counted from counts per minute (CPM) measured on scintillation counter LS100C (Beckman Coulter, Brea, CA, USA).

### 4.5. EMSA (Electrophoretic Mobility Shift Assay)

EMSA was held with different radiolabeled RNAs: 5′UTR of potato virus Y [38] and its variations (‘I’, ‘720’, ‘C’, ‘F’), 5′UTR of potato virus M (NC_001361.2), 5′UTR of tobacco mosaic virus (Ω-element) [39], 5′UTR of EcCspA [40], epsilon element εII [25], and 5′UTR of tobacco etch virus (TEV) [41]. All these probes were synthesized from the same pBluescriptII matrices as for *in vitro* translation but linearized by *Nco*I. Radiolabeled mRNAs (1 μg) were used for the binding reactions in a 10 μL volume containing 10 mM Tris, pH 7.5, 35 mM KCl, 1.0 mM MgCl_2_, 5% glycerol, 1 mM DTT, and 1 units/μL RNasin. The proteins AtUBP1b and BSA were added at a 5:1 ratio to RNA. The procedure was performed according to [42]. RNA visualization after incubation with proteins was performed on native 10% PAGE in TAE with subsequent gel drying and X-film CP-BU-M (Agfa, Mortsel, Belgium) exposition for 5 days.

### 4.6. Western Blotting for Determination of Protein Phosphorylation

To detect protein phosphorylation, Western blotting was performed with antibodies against the phospho-forms of the amino acids serine and threonine (Santa Cruz Biotechnology, Dallas, TX, USA). Denaturing electrophoresis was performed according to [43], 5 μg of protein was applied to each lane, and transfer to a PVDF membrane (Thermo Fisher Scientific, Waltham, MA, USA) was performed by semi-dry blotting. The membrane was loaded in TBS with 2% BSA for 1 h with gentle agitation at RT. Monoclonal mouse antibodies against phospho-Ser were used in 1:200 dilution; monoclonal mouse antibodies against phospho-Thr conjugated with HRP were used in 1:150 dilution. For further phospho-Ser detection we used secondary antibodies against the whole mouse IgG produced in goat, which was conjugated with HRP (Santa Cruz Biotechnology, Dallas, TX, USA); it was diluted 1:2000. For HRP detection we used Chemiluminescent Peroxidase Substrate-3 (Sigma-Aldrich, St. Louis, MO, USA) with subsequent exposure on X-film CP-BU-M (Agfa, Mortsel, Belgium) or a colorimetric metal-enhanced DAB substrate kit (Thermo Fisher Scientific, Waltham, MA, USA).

### 4.7. Protein Dephosphorylation by λ-Phosphatase

Proteins AtUBP1b and BSA (as negative control) were dephosphorylated with lambda-phosphatase (Santa Cruz Biotechnology, Dallas, TX, USA). Then, 10 nmoles of proteins was added to a 50 μL volume containing 50 mM HEPES, pH 7.5, 0.1 mM EGTA, 5 mM DTT, 0.01% BRIJ 35, 2 mM MnCl_2_, and 2000 units of λ-phosphatase. The reaction mixture was incubated for 1 h at 30 °C; the reaction was stopped by freezing at −80 °C.

### 4.8. Liquid Chromatography (LC)-MS Analysis

Sample preparation for mass spectrometry was as follows. Bands of desired size were cut from the polyacrylamide gel (stained with Coomasie G-250) and dehydrated in 100% acetonitrile for 15 min at room temperature. Thereafter dehydrated gel pieces were submerged in trypsin solution (50 mM Hepes, pH 7.6, 0.5 M urea, and 1 µg trypsin) and incubated for 16 h at 37 °C. After incubation, the supernatant was collected, acidified with formic acid (final concentration, 3%), and cleaned by reversed phase solid phase extraction (STRATA-X; Phenomenex, Torrance, CA, USA). After drying, samples were dissolved in 20 µL of 3% acetonitrile and 0.1% formic acid.

For the LC-MS run, the auto sampler Ultimate 3000 RSLC system (Thermo Fisher Scientific, Waltham, MA, USA) was used with the C18 guard desalting column (Thermo Fisher Scientific, Waltham, MA, USA). Total LC-MS run time was 75 min. We used a nano EASY-Spray column (Thermo Fisher Scientific, Waltham, MA, USA) on the nano electrospray ionization EASY-Spray source (Thermo Fisher Scientific) at 60 °C. Online LC-MS was performed using a hybrid Q-Exactive HF mass spectrometer (Thermo Fisher Scientific, Waltham, MA, USA). Dynamic exclusion was used with 30 s duration. An underfill ratio of 1% was used.

### 4.9. Proteomics Database Search

All MS/MS spectra were searched with the software Mascot search engine (Matrix Science) using a target-decoy strategy. The reference database was the SwissProt 2025_01 (572,970 sequences) with taxonomy of *Arabidopsis thaliana* (16,396 sequences). Additional settings included trypsin/*p* (cuts C-term side of KR), with such variable modifications as oxidation of methionine, acetylation on the protein N-terminus, and phosphorylation on serine, threonine, and tyrosine. Peptides found at 1.01% false discovery rate (FDR) were used by the protein grouping algorithm.

### 4.10. Statistical Analysis

To determine the significance degree of differences between the means of control and the experimental data for *in vitro* translation results, the Student’s *t*-test was used; 5% level of significance was accepted (α ≤ 0.05). The actual *t*-test for each compared dataset was calculated as an independent two-sample *t*-test with unequal variances and two-sided distribution using the Microsoft Excel function “TTEST”. The statistical test power was calculated in STATA 13 by the “test power” operator. We calculated the effect size for all tests performed. For comparison of two mean values, we used Cohen’s *d* as an effect size measurement (*d* value of 0.2 indicated a small effect, 0.5 indicated a medium-sized effect, and 0.8 indicated a large effect) [44].

## 5. Conclusions

Our data indicate the following facts: (1) AtUBP1b protein binds to mRNA specifically depending on its sequence (secondary structure); (2) AtUBP1b protein is phosphorylated at serine, threonine, and tyrosine residues in *E. coli* cells, and protein phosphorylation also occurs in S23 and S100 extracts from wheat germ; (3) addition of AtUBP1b into the *in vitro* protein translation system causes inhibition of protein synthesis for those mRNAs which contain 5′UTR that is able to bind AtUBP1b; (4) the ability of AtUBP1b protein to bind mRNA might depend on the protein phosphorylation, but the effect of the latter (positive or negative) stays unclear.

## Figures and Tables

**Table 1:** Sequences of 5′UTR used for gel-shifting assay.

UTR	Sequence	U Content,%
5′PVY	****AAUUAAAACAACUCAAUACAACAUAAGAAAAACAACGCAAAAACACUCAUAAACGCUUAUUCUCA****CUCAAGCAACUUGCUAAGUUUCAGUUUAAAUCAUUUCCUUGCAACUCUCUUAAACGAUAUUGGAAAC****CAUUUCAACUCAACAAGUAAUUUCAUCACUUCCAACCAAUUUCAGAUCCU ^1^ (182 nt)	28%
5′CspA	GGUUUGACGUACAGACCAUUAAAGCAGUGUAGUAAGGCAAGUCCCUUCAAGAGUUAUCGUUGAUACCCCUCGUAGUGCACAUUCCUUUAACGCUUCAAAAUCUGUAAAGCACGCCAUAUCGCCGAAAGGCACACUUAAUUAUUAAAGGUAAUACAC (156 nt)	26%
5′TEV	AAAUAACAAAUCUCAACACAACAUAUACAAAACAAACGAAUCUCAAGCAAUCAAGCAUUCUACUUCUAUUGCAGCAAUUUAAAUCAUUUCUUUUAAAGCAAAAGCAAUUUUCUGAAAAUUUUCACCAUUUACGAACGAUAGCA (143 nt)	28%
5′I	UAAUUAAAACAACUCAAUACAACAUAAGAAAAACAACGCAAAAACACUCAUAAACGCUUAUUCUCACUCAAGCAACUUGCUAAGUUUCAGUUUAAAUCAUUUCCUUGCAACUCUCUUAAACGAUAUUGGAAA (132 nt)	27%
5′Ω	GACCAUGAUUAGGGGGGAGAUUUAUUUUUACAACAAUUACCAACAACAACAAACAACAAACAACAUUACAAUUACUAUUUACAAUUACAGUCGACGAU (98 nt)	26%
5′PVM	AUUAAACAAACAUACAAUAUCUGGACUUACACUACAAUAUACUACCAGGAAAUACUAUAUUUGGUCUAAGUCAGC (75 nt)	28%
5′F	UAAUUAAAACAACUCAAUACAACAUAAGAAAAACAACGCAAAAACACUCAUAAACGCUUAUUCUCACUCA (70 nt)	20%
5′C	CUCAAGCAACUUGCUAAGUUUCAGUUUAAAUCAUUUCCUUGCAACUCUCUUAAACGAUAUUGGAAAC (67 nt)	34%
5′720	CAUUUCAACUCAACAAGUAAUUUCAUCACUUCCAACCAAUUUCAGAUCCU (50 nt)	32%
5′εII	UCUAGAUUUAACUUUAUUUACCUUAUCUCUAUUCCAUGG (39 nt)	49%

^1^ The following elements are shown in the 5′PVY sequence: 5′I (bold), 5′F (double underline), 5′C (dashed), 5′720 (underline).

## Data Availability

The original contributions presented in the study are included in the article. All the data included in this article are publicly available. Further inquiries can be directed to the corresponding author.

## Supplementary Materials

The following supporting information can be downloaded at: https://www.mdpi.com/article/10.3390/plants14203191/s1, Figure S1: Western blot of AtUBP1b protein isolated from *E. coli* cells without addition of phosphatase inhibitors; Table S1: Statistical analysis of *in vitro* translation results; Figure S2: Secondary structures of RNA 5′UTR obtained in RNAstructure ver.5.4 (Mathews Lab).

## Abbreviations

The following abbreviations are used in this manuscript:

SGs	Stress granules
5′TOP	5′-terminal oligopyrimidine tracts
AtUBP1b	Oligouridylate binding protein
UTR	untranslated region
EMSA	electrophoretic mobility shift assay
BSA	bovine serum albumin
GUS	β-glucuronidase
PVY	potato virus Y
PVM	potato virus M
TMV	tobacco mosaic virus
EcCspA	cold-shock protein A of *E. coli*
TEV	tobacco etch virus
RRM	RNA recognition motifs
