# Supplementary material for: Study of Stress Granule Core Protein AtUBP1b Phosphorylation In Vitro
Source: Plants (Basel). 2025 Oct 17;14(20):3191. doi: 10.3390/plants14203191 (PMC12567214; doi:10.3390/plants14203191)
Supplement: Supplementary file 1 [file plants-14-03191-s001.zip › plants-3878876-supplementary.pdf]

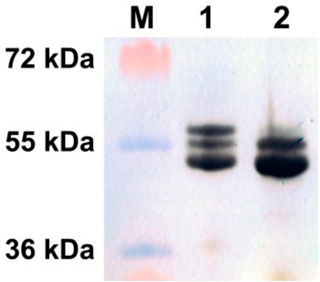

Figure S1. Western blot of AtUBP1b protein isolated from *E. coli* cells without addition of phosphatase inhibitors. Lanes: 1 – crude lysate of total cellular protein; 2 – purified AtUBP1b, dialyzed twice against TBS after purification; M – protein marker Page Ruler Prestained Protein Ladder Plus (“Fermentas”).

Table S1 – Statistical analysis of *in vitro* translation results.

Statistical parameters	‘BSA+λ’ to ‘BSA’	‘UBP+λ’ to ‘UBP’	‘UBP+λ’ to ‘BSA+λ’	‘UBP’ to ‘BSA’
Ω-GUS-TMV (n=5 for ‘BSA+λ’ and ‘UBP+λ; n=4 for the rest)				
Significance level (α)	0.544	0.215	0.001	0.031
Power (1-β)	0.560	0.217	0.923	0.908
d	0.196	0.063	2.980	2.418
ratio (%)	1.063	1.339	0.302	0.332
PVY-GUS-PVY (n=5)				
Significance level (α)	0.077	0.023	0.001	0.004
Power (1-β)	0.222	0.299	0.998	0.868
d	0.625	1.085	4.097	2.525
ratio (%)	0.807	0.585	0.201	0.278
TEV-GUS-TMV (n=5)				
Significance level (α)	0.111	0.000	0.028	0.005
Power (1-β)	0.212	0.063	0.947	0.999
d	0.482	1.221	2.418	3.896
ratio (%)	0.843	0.749	0.226	0.254
720-GUS-PVY (n=4)				
Significance level (α)	0.190	0.140	0.375	0.714
Power (1-β)	0.365	0.762	0.308	0.127
d	0.246	0.349	0.416	0.718
ratio (%)	1.087	1.208	1.082	0.973

Parameters that have statistical significance/reliability are shaded in gray. Significance level (α) was determined as two-sided paired t-test; the level of statistical significance was $\alpha \leq 0.05$. Power of the test (1-β) was taken as 80%. Cohens *d* (standardized effect size) was interpreted as large effect size at $d \geq 0.8$. Ratio (%) shows the multiple of the value of one sample in relation to another (e.g. ‘UBP+λ’ / ‘UBP’).

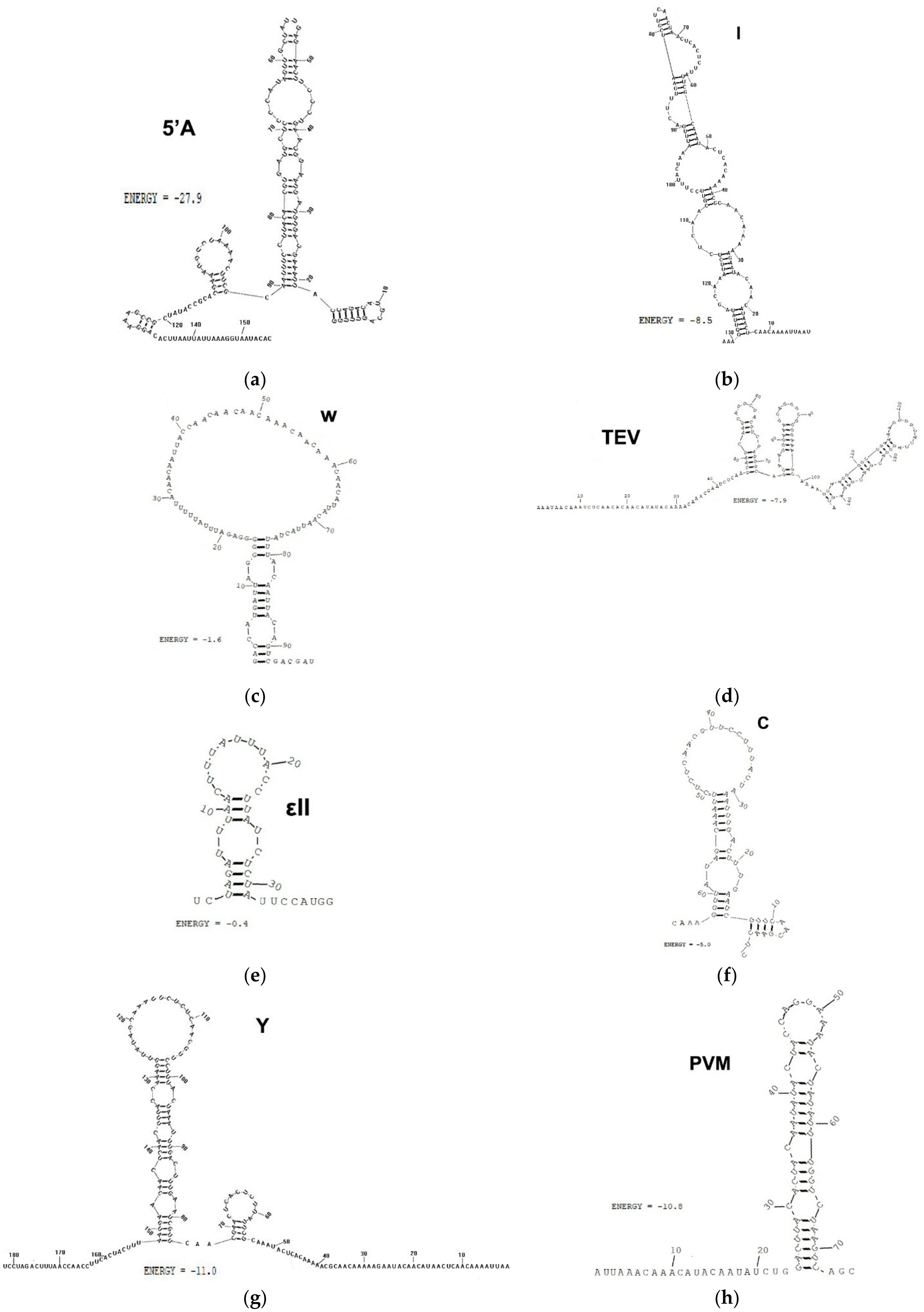

Figure S2. Secondary structures of RNA 5′UTR obtained in RNAstructure ver.5.4 (Mathews Lab). (**a**) 5′CspA; (**b**) 5′I; (**c**) Ω; (**d**) 5′TEV; (**e**) εII (ipsilon); (**f**) 5′C; (**g**)

5′PVY; (**h**) 5′PVM. Indications in bold letters on the figures correspond the gel lanes on the Figure 5.
